# Epidemiological Investigation and Prevention Control Analysis of the Longitudinal Distribution of COVID-19 in Henan Province, China

**DOI:** 10.1128/mSphere.00867-20

**Published:** 2020-10-07

**Authors:** Xianguang Yang, Xuelin Chen, Cuihong Ding, Zhibo Bai, Jingyi Zhu, Gege Sun, Guoying Yu

**Affiliations:** a College of Life Science, Henan Normal University, Xinxiang, Henan Province, China; U.S. Centers for Disease Control and Prevention

**Keywords:** COVID-19, Henan Province, epidemic, prevention and control

## Abstract

The objective was to analyze the longitudinal distribution, epidemiological characteristics, and local prevention and control measures of coronavirus disease 2019 (COVID-19) in six cities in Henan Province, China, from 21 January 2020 to 17 June 2020: Xinyang City (including Gushi County), Nanyang City (including Dengzhou City), Zhumadian City (including Xincai County), Zhengzhou City (including Gongyi City), Puyang City, and Anyang City (including Hua County). Data were collected and analyzed through the COVID-19 information published on the official websites of the health commissions in the six selected cities of Henan Province. As of 17 June 2020, the cumulative incidence rate of COVID-19 in Henan Province was 1.33/100,000, the cumulative cure rate was 98.27%, the cumulative mortality rate was 1.73%, the age range of diagnosed cases was 5 days to 85 years old, and the male-to-female ratio was 1.09:1. The confirmed cases of COVID-19 in Henan Province were mainly imported cases from Hubei, accounting for 87.74% of all cases, of which the highest proportion was 70.50% in Zhumadian. The contact cases and local cases increased in a fluctuating manner over time. In this paper, epidemiological characteristics of COVID-19 in Henan Province were analyzed from the onset of the outbreak to the effective control within 60 days, and effective and distinctive prevention and control measures in various cities were summarized to provide a favorable useful reference for the further formulation and implementation of epidemic prevention and control and a valuable theoretical basis for effectively avoiding a second outbreak.

**IMPORTANCE** Epidemic prevention and control in China have entered a new stage of normalization. This article analyzes the epidemiological characteristics of COVID-19 in Henan Province and summarizes the effective disease prevention and control means and measures at the prefecture level; the normalized private data provide a theoretical reference for the formulation and conduct of future prevention and control work. At the same time, these epidemic prevention and control findings can also be used for reference in other countries and regions.

## Abstract (precis)

Epidemic prevention and control in China have entered a new stage of normalization. This article analyzes the epidemiological characteristics of COVID-19 in Henan Province and summarizes the effective disease prevention and control means and measures at the prefecture level; the normalized private data provide a theoretical reference for the formulation and conduct of future prevention and control work. At the same time, these epidemic prevention and control findings can also be used for reference in other countries and regions.

## INTRODUCTION

In December 2019, Wuhan, Hubei Province, China, reported a number of cases of viral pneumonia of unknown cause (1); since then, the rapid spread of this disease has aroused great concern from society as a whole. The virus was confirmed to be a novel coronavirus with a unique genome by sequencing the lower respiratory tract samples collected by an expert group. The novel virus was initially named 2019 novel coronavirus (2019-nCoV-2); it was later designated by the International Committee for the Classification of Viruses as severe acute respiratory syndrome coronavirus 2 (SARS-CoV-2). The novel virus SARS-CoV-2 spread throughout the world within 3 months, causing a pandemic and posing a serious threat to human health (2). On 11 February 2020, the World Health Organization (WHO) officially named this pneumonia coronavirus disease 2019 (COVID‐19) and declared the infectious disease a public health emergency (3). The SARS-CoV-2 virus is reported to have spread through human-to-human transmission (4).

To date, there is a lack of population-level immunity against COVID-19 in humans (5). At present, the known cause of COVID-19 infection is SARS-CoV-2 infection (including asymptomatic infection), which is transmitted by respiratory droplets, close contact, aerosols, and fecal-oral transfer (6, 7). Respiratory droplets and close contact are the main transmission routes, and in general, most people are susceptible. Based on the epidemiological investigations to date, the incubation period of this disease is 1 to 14 days (most often 3 to 7 days). Fatigue, dry cough, and fever have been the main symptoms of most infected persons. A small number of patients have also had nasal congestion, runny nose, sore throat, diarrhea, and other symptoms. Computed tomography (CT) examination showed chest imaging characteristics. Most of the patients with severe illness have developed dyspnea or/and hypoxemia 7 days after onset. Critically ill patients may rapidly progress to acute respiratory distress syndrome, septic shock, multiple organ failure, and other symptoms. It should be noted that most of the patients with severe and critical diseases have had moderate to low fever or no obvious fever symptoms (8–10).

Henan Province is adjacent to Hubei Province, with Xinyang, Nanyang, and Zhumadian as the border cities. Zhengzhou, the capital city of Henan Province, has a large flow of people, so the four cities are high-COVID-19-incidence areas in Henan Province. In this paper, 6 cities were selected according to their geographic distribution from south to north in Henan Province (south: Nanyang, Xinyang, and Zhumadian; central: Zhengzhou City; north: Anyang and Puyang) for an epidemiological investigation of the COVID-19 outbreak and analysis of the prevention and control measures to inform the ongoing epidemic prevention and control work, prevent a secondary outbreak, and provide a theoretical basis for the disease (11).

## RESULTS

### Basic epidemiological characteristics of COVID-19 in Henan Province.

**FIG 1:**
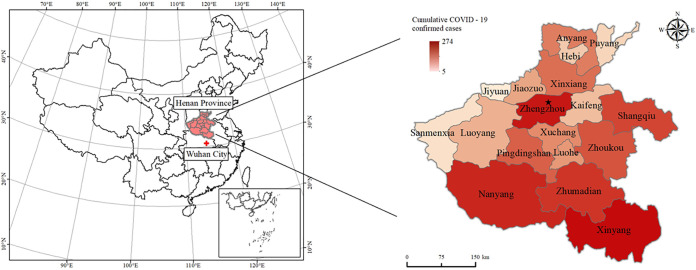
Distribution of COVID-19 in Henan Province, China. The number at top indicates the cumulative confirmed COVID-19 cases in Henan Province, China, until 17 June 2020. The different shades represent the overall incidence of confirmed COVID-19 cases within the general population within each city of Henan Province, China. The star stands for Zhengzhou, the capital of Henan Province.

**(i) The basic situation of the epidemic in Henan Province.** Since the first confirmed imported case was reported in Zhengzhou on 21 January 2020, the daily number of newly confirmed cases in the province has fluctuated with time. On 4 February 2020, the province’s number of new confirmed cases reached a peak number of 109 people Since then, the entire province’s daily total number of new confirmed cases fluctuated and declined over time. On 24 February 2020, Henan Province had, for the first time, no new confirmed cases; on 11 March 2020, 1,272 confirmed imported cases of COVID-19 were reported in Zhengzhou due to outside input (from Italy). A total of 1,273 confirmed COVID-19 cases have been reported in Henan Province as of 17 June 2020, and the distribution of COVID-19 cases in each city is shown in Fig. 1. The cumulative incidence was 1.33 per 100,000.

On 29 January 2020, the first cured and discharged COVID-19 case was reported in Henan Province. From 2 February to 8 March, the total number of COVID-19 cured and discharged cases in Henan Province rose with time, reaching a peak of 103 cases on 20 February. From 9 to 21 March 2020, 1 new COVID-19 patient was cured and discharged from the hospital. By 19 March, the number of confirmed COVID-19 hospitalized cases in the province had been reduced to zero; a total of 1,250 COVID-19 patients had been cured and discharged from the hospital, with a cure rate of 98.19%.

**FIG 2:**
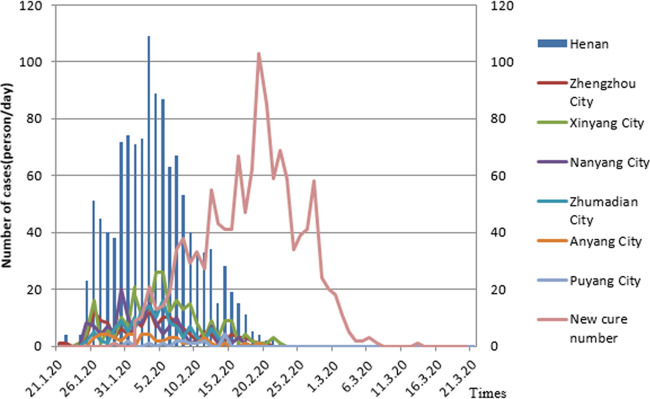
Daily statistics of the epidemic in Henan Province.

On 26 January 2020, there was one deceased case of COVID-19; this was the first deceased case in Nanyang, Henan Province. By 21 March, 2020, there had been 22 COVID-19 deaths in Henan Province, with a fatality rate of 1.73%. There were 1,148 mild cases in the province, accounting for 90.25% of the total cases. There were 64 severe cases, accounting for 5.03%. The total number of critical cases was 38, accounting for 2.99% (Fig. 2) of the total cases.

**FIG 3:**
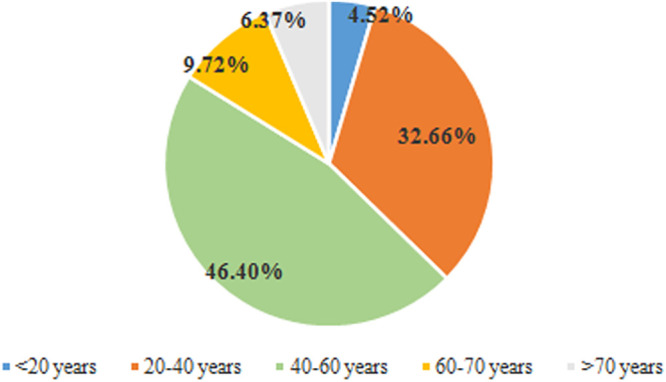
Age ratio of confirmed COVID-19 cases in Henan Province. Here is a breakdown of age structure: <60 years old, 20 years per age period; >60 years old, 10 years per age period.

**(ii) Basic characteristics of confirmed cases in Henan Province.** Among the 600 confirmed cases, 315 were male and 285 were female, with a sex ratio of 1.11 to 1. The ages of the confirmed cases ranged from 5 days to 85 years; the oldest case was from Zhumadian, and the youngest was from Xinyang. The average age of the confirmed cases was 45 years, the median age was 46 years, and the age mode was 56 years. Therefore, while most of the population was generally susceptible to COVID-19, the middle-aged and elderly populations (aged 40 to 60 years) were more likely to be infected (Fig. 3). Among the 600 confirmed COVID-19 cases, 204 were contact cases, accounting for 34% of the total number of confirmed cases, and 73 were local cases, accounting for 12.17% of the total number of confirmed cases. Among them, 325 were imported cases, of which 277 were imported cases in Hubei Province, accounting for 85.23% of imported cases.

### COVID-19 epidemic situation at the municipal level.

**(i) Variations in the COVID-19 trends in each city.** As of 24:00 on 17 June 2020, a total of 157 confirmed COVID-19 cases had been reported in Zhengzhou City, with a cumulative incidence of 142/100,000. Xinyang City reported 274 confirmed COVID-19 cases, with a cumulative incidence of 3.61/100,000. In Nanyang, 156 confirmed COVID-19 cases have been reported, with a cumulative incidence rate of 1.35 per 100,000. In Zhumadian, 139 confirmed COVID-19 cases have been reported, with a cumulative incidence of 175/100,000. Anyang City has reported 53 confirmed cases of COVID-19, with a cumulative incidence of 0.8 per 100,000. Puyang City has reported a total of 17 confirmed COVID-19 cases, with a cumulative incidence of 0.5 per 100,000. These six cities accounted for 62.26% of the province’s total number of confirmed COVID-19 cases. Xinyang had the highest incidence of COVID-19, followed by Zhumadian, Zhengzhou, Nanyang, and Anyang City. Puyang City had the lowest incidence of COVID-19. According to the data, the severity of COVID-19 decreased from north to south according to the geographical location and movement of people in various cities in Henan Province. As the capital city of Henan Province, Zhengzhou has a complex population, and a large number and wide range of people move in and out of this city. The overall COVID-19 epidemic burden was higher in Zhengzhou than in other central regions. On 23 February 2020, Xinyang City had no new confirmed COVID-19 cases for the first time, and Henan Province had no newly confirmed COVID-19 cases for the first time on 24 February. By 10 March 2020, a total of 1,272 confirmed COVID-19 cases had been reported in Henan Province, and a confirmed imported COVID-19 case was found in Zhengzhou on 11 March. Since then, no new confirmed COVID-19 cases have been reported in Henan Province; as of 17 June 2020, there have been a total of 1,273 confirmed COVID-19 cases.

**(ii) Age distribution and sex ratio of the confirmed cases.** The male-to-female ratio of confirmed COVID-19 cases was 1.38:1 in Xinyang, 0.78:1 in Nanyang, 1.58:1 in Zhumadian, 1.14:1 in Zhengzhou, and 0.55:1 in Puyang (Anyang City disclosed only the number of confirmed cases and did not disclose the detailed information of confirmed cases, so the following were characteristics were not analyzed in detail for this city). According to the analysis of the sex ratio among cities, the cumulative incidence of COVID-19 among males in Zhengzhou, Xinyang, and Zhumadian was higher than that among females. Among these cities, the cumulative incidence of COVID-19 among males in Xinyang was the highest (4.08 per 100,000). Puyang had the lowest incidence of COVID-19 among males (0.34/100,000). The cumulative incidence of COVID-19 among women in Nanyang and Puyang was higher than that of men, and the highest incidence of COVID-19 among women in was in Xinyang (3.11/100,000), while the lowest incidence of COVID-19 among women was in Puyang (1.33/100,000).

For the age analysis, to make a correct and objective analysis of the age composition structure, age was divided according to the age structure in the 2019 *Henan Statistical Yearbook*. The age of confirmed COVID-19 cases ranged from 5 days (Xinyang) to 88 years (Zhumadian), and the age of confirmed COVID-19 cases was concentrated between 15 and 64 years, accounting for 87.42%. The cumulative incidence of COVID-19 in this age group was the highest (511/100,000) in Xinyang. The cumulative incidence of COVID-19 at ages ≤14 years was the lowest, with Nanyang having the lowest incidence (0.10/100,000). The results are shown in Table 1. The average age of confirmed COVID-19 cases was 42.8 years in Zhengzhou City, 47.3 years in Xinyang, 44.4 years in Nanyang, 43.2 years in Zhumadian, and 39.5 years in Puyang.

**TABLE 1:** Longitudinal analysis of confirmed cases and cumulative incidence of COVID-19 in six cities in Henan Province as of 17 June 2020^a^^,^^b^

Patients	Xinyang City		Nanyang City		Zhumadian City		Zhengzhou City		Puyang City		Anyang City	
	Conf. cases [*n* (%)]	Cumul. incid. (1/100,000)	Conf. cases [*n* (%)]	Cumul. incid. (1/100,000)	Conf. cases [*n* (%)]	Cumul. incid. (1/100,000)	Conf. cases [*n* (%)]	Cumul. incid. (1/100,000)	Conf. cases [*n* (%)]	Cumul. incid. (1/100,000)	Conf. cases [*n* (%)]	Cumul. incid. (1/100,000)
Total	274 (100.0)	3.61	156 (100.0)	1.35	139 (100.0)	1.75	157 (100.0)	1.42	17 (100.0)	0.5	53 (100.0)	0.8
Male patients	159 (58.03)	4.08	67 (42.95)	1.17	84 (60.43)	2.13	83 (52.86)	1.5	6 (35.29)	0.34	—	—
Female patients	115 (41.97)	3.11	86 (55.13)	1.50	53 (38.13)	1.46	73 (46.49)	1.34	11 (64.71)	1.33	—	—
Missing data	0	—	3 (1.92)	—	2 (1.44)	—	1 (0.65)	—	0	—	—	—
≤14 yr old	5 (1.82)	0.30	3 (1.92)	0.10	5 (3.60)	0.28	6 (3.82)	0.28	1 (5.88)	0.11	—	—
15–64 yr old	246 (89.78)	5.11	132 (84.62)	1.84	120 (86.33)	2.40	134 (85.35)	1.77	14 (82.35)	0.6	—	—
≥65 yr old	23 (8.39)	2.02	18 (11.54)	1.27	14 (10.07)	1.28	16 (10.19)	1.3	2 (11.76)	0.49	—	—
Missing data	0	—	3 (1.92)	—	0	—	1 (0.64)	—	0	—	—	—

a This table presents only statistics from the official website, and the age structure is based on the ages in the *Statistical Yearbook* of Henan Province.

b Abbreviations and symbol: Conf., confirmed; Cumul. incid., cumulative incidence; —, data missing or not statistically significant.

**(iii) Source of confirmed cases.** According to the data, the main confirmed COVID-19 case source was imported cases, among which the highest proportion of imported cases was 70.50% in Zhumadian, followed by Zhengzhou. Xinyang City had the highest total number of confirmed COVID-19 cases (273 cases). Imported cases accounted for 134 cases, accounting for 49.08% of the total confirmed cases of COVID-19. The results are shown in Table 2. According to the public epidemic notification information recorded in Henan Province, confirmed imported cases were selected from Xinyang, Zhengzhou, Zhumadian, and Nanyang City for detailed investigation, and the imported cases were subdivided into confirmed imported cases from Hubei sources and cases not imported from Hubei sources. The results are shown in Table 3. Hubei accounted for the majority (87.74%) of the total imported COVID-19 cases.

**TABLE 2:** Source types for confirmed COVID-19 cases^*a*^

Case type	Xinyang City		Nanyang City		Zhumadian City		Zhengzhou City		Puyang City		Anyang City	
	No. of confirmed cases	%	No. of confirmed cases	%	No. of confirmed cases	%	No. of confirmed cases	%	No. of confirmed cases	%	No. of confirmed cases	%
Imported cases	134	49.08	85	55.19	98	70.50	99	63.06	6	35.29	—^*b*^	—
Contact cases	110	40.29	53	34.42	29	20.86	33	21.02	7	41.18	—	—
Local cases	29	10.62	15	9.74	12	8.63	25	15.92	4	23.53	—	—
Missing data	—	—	1	0.65	—	—	—	—	—	—	—	—

a Note that this table presents only statistics from the official website, and the ages in the structure are based on the ages in the *Statistical Yearbook* of Henan Province.

b —, data missing or not statistically significant; for missing data, details of the confirmed cases are unknown.

**TABLE 3:** Analysis of imported cases in areas with high epidemic incidence in Henan Province

Importation status	Confirmed cases [*n* (%)]			
	Xinyang City	Zhengzhou City	Zhumadian City	Nanyang City
Imported cases from Hubei	128 (46.72)	69 (43.95)	92 (66.19)	76 (49.03)
Cases not imported from Hubei	9 (3.28)	32 (20.38)	6 (4.32)	4 (2.58)

### Epidemic prevention and control. (i) National epidemic prevention and control.

To do a good job in terms of the prevention and control of the COVID-19 epidemic in China and safeguard the health and safety of the people, the National Health Commission has drawn up a novel Coronavirus Pneumonia Prevention and Control Plan to prevent the spread of the epidemic in light of the epidemic situation and the latest research progress. The health administration departments at all levels are required to coordinate with the Chinese Center for Disease Control and Prevention (CDC), allocate funds and materials for epidemic prevention and control in a timely manner, carry out joint prevention and control work, identify and report cases quickly, and provide timely feedback and analysis. Medical institutions at all levels and of various types are responsible for the detection, reporting, isolation, diagnosis, treatment, and clinical management of cases. While preventing and treating cases, these institutions also care for people’s mental health problems and do a good job in health education and risk communication to the public.

**(ii) Main prevention and control measures of the Henan Provincial Government.** In the face of COVID-19, the Henan Provincial Government responded positively, quickly implemented the guidance given in the document of the Communist Party of China (CPC) Central Committee, and added a number of important prevention and control measures specific to the Henan characteristics. First, publicity was increased. The government has made the public aware of the seriousness of COVID-19 through TV and radio, 24 h a day and 7 days a week, and sent text messages on epidemic prevention and control to the public through mobile phone platforms. Second, supervision was strengthened. Since the outbreak of the epidemic, Henan Province has given full play to big data computing. The “Health Code” of Henan Province has been recognized by 31 provinces, autonomous regions, and municipalities, and 13.98 million people have applied for it. Data on the movement of citizens are recorded in the cloud, including real-name records (streets, bring, village, etc.). Grassroots community units have been tasked with strict screening of Hubei Province, including Xinyang, Nanyang, Zhumadian, and Zhengzhou, which are areas that were seriously affected by COVID-19. These units have been instructed to take the necessary actions to monitor home quarantine for at least 14 days, stop all provincial traffic operations, provide public supervision and prevention, control the epidemic reporting platform, and encourage the populace to report exposures and cases. Third, the epidemic was fought comprehensively. All units at all levels of the health care system canceled the Spring Festival holiday in 2020, and all staff arrived at their posts on time to maintain 24-h communications and respond to the epidemic at any time; 130 hospitals were identified and designated for the timely medical treatment of COVID-19.

**(iii) Specific epidemic prevention and control measures.** All cities in Henan Province promptly launched the first-level response for major health emergencies and adopted epidemic prevention and control measures specific to the characteristics of each city, particularly the high-COVID-19-incidence areas in Henan Province (Zhengzhou, Xinyang, Nanyang, and Zhumadian City). As an example, Zhengzhou opened a special information line that provides 24-h free COVID-19 services; the city also stopped all travel services from outside Zhengzhou and reduced the city public traffic divisions. Residents of each district area had a yard code registration management system that tracked those not obeying the compulsory isolation. Ten card swipe points were set up in Xinyang, and the vehicle traffic was checked at the same time for full sanitization. Temperature monitoring was carried out by the staff to ensure that no one in the car had a fever, and vehicles and personnel coming from the direction of Hubei were advised to return to the territory of Xinyang. The staff ensured that patients with fever were reported to the epidemic prevention and control command in a timely manner and arranged for further isolation. All entertainment venues were closed for business. No food provided by the catering industry, whether for a large or small gathering, was allowed to enter public places. Only when a person had no symptoms could the person enter public places with a mask and a temperature measurement. Nanyang postponed the work of government organizations, the resumption of work of various enterprises, the opening of schools, and the opening time of administrative examination and approval services; the Nanyang authorities also implemented a centralized isolation and observation system and adopted reward-linked reporting measures to encourage citizens to supervise those around them to control the epidemic in an all-round and multifaceted way. Zhumadian and its subsidiaries closed four fire stations, suspended all traffic operations, and closed farmers’ markets and entertainment venues. In addition to the above four cities, all other cities have imposed compulsory control measures on the traffic operations within the city, including subordinate counties, and suspended all kinds of public service places at all levels as well as all kinds of external purchase and distribution activities, to effectively ensure internal nonproliferation and the prevention of external import.

## DISCUSSION

COVID-19 is a new and highly infectious disease that emerged in 2020. It has had a great impact on the whole society. It seriously affects not only people’s physical and mental health but also people’s productivity and lives. Henan Province, which is close to Hubei Province, is a province with a large population and high degree of resident mobility. As of 17 June 2020, Henan Province had a total of 1,273 confirmed cases, with a cumulative incidence rate of 1.32/100,000, which was higher than that of other provinces and cities outside Hubei and closely related to the geographical location of Henan Province. Since the first confirmed case was reported in Henan Province, the number of newly confirmed cases per day generally showed an inverted V-shaped trend, reaching a peak of 109 cases on 20 February, and the epidemic reached stable control after 24 February; after that point, there were no new cases except one case imported from abroad on 11 March. The study shows that the population migration and COVID-19 cumulative disease rate are positively correlated, Zhengzhou is the capital city and the main transport hub city of Henan Province; the population is largely fluid. Zhumadian City has 4 quick ways to access Wuhan—Nanyang, Xinyang, and Nanyang City in Henan Province and the junction with Hubei Province; the main direction of travel is from Hubei Province. Due to convenient transportation and a drive time of only 30 min, Hubei Province is the first choice for migrant workers and entertainment, so the above four cities of Henan Province have had COVID-19 outbreaks. Anyang and Puyang cities are in the northernmost part of Henan Province, far from Hubei Province, so these areas were less affected by the epidemic (12).

In terms of the source of confirmed COVID-19 cases, Hubei-imported cases dominated the initial stage of the outbreak. Contact cases and local cases fluctuated over time, indicating that the outbreak shifted from external import to internal spread. However, the total proportion of contact cases and local cases was 42.80%, indicating that there was no large-scale spread of the epidemic in Henan Province. It is worth noting that one imported case occurred in Zhengzhou on 11 March, and the confirmed imported cases from central Hubei accounted for 20.38% of the total cases in Zhengzhou City. Therefore, Zhengzhou City should increase travel from other non-Hubei and foreign regions while preventing the import of cases from Hubei.

Studies by the COVID-19 Epidemiology Group of the Chinese Center for Disease Control and Prevention showed that the sex ratios of confirmed COVID-19 cases in China, Hubei Province, and Wuhan were 1.06:1, 1.04:1, and 0.99:1, respectively (13). The studies by Zhong Nanshan showed that female patients accounted for 41.9% of the total group of patients (14); in Xi’an, the Center for Disease Control and Prevention reported that male patients accounted for 54.3% of the total cases (Yao et al., 2020 [15]), and Wang et al. (16) and Kang et al. (17) confirmed that women were more prevalent than men among the cases in Guangzhou. While prompt COVID-19 detection and COVID-19 risk were similar between men and women, the sex distributions of the confirmed cases were different in different areas. For example, there were more male patients than female patients in Zhengzhou City, Xinyang, and Zhumadian City while there were more female patients than male patients in Puyang and Nanyang City.

Since 21 January, the province has reported 1,273 confirmed COVID-19 cases (including one imported from abroad) and 22 deaths. As the last COVID-19 patient in Xinyang was discharged from the hospital on 14 March, all local hospitalized patients in the province were cured and discharged, and there were zero cases on 15 March. The rapid epidemic prevention measures not only effectively controlled the source of infection but also cut off the transmission route of SARS-CoV-2, so that the outbreak could be quickly brought under control in a short time. At present, the COVID-19 epidemic in our province has been effectively controlled, but officials are cautioning residents not to relax their vigilance. With the return of overseas students and the return of overseas staff, we should strictly control the import of external cases, strictly control the risk of imported cases, strengthen epidemic prevention and control work in our province, and earnestly achieve the goal of “internal anti-diffusion, external anti-input” while preventing the spread of asymptomatic infected persons in our province.

## MATERIALS AND METHODS

### Data sources.

The number of permanent residents in Henan Province and the 6 selected cities (including counties) in this paper were determined according to the Third Chapter of the 2019 Henan Statistical Yearbook (http://oss.henan.gov.cn/sbgt-wztipt/attachment/hntjj/hntj/lib/tjnj/2019/zk/indexch.htm). According to the official website of the Henan Provincial Health Commission, “The latest situation of COVID-19 in Henan Province” (http://wsjkw.henan.gov.cn/2020/06-18/1551107.html), the overall data from the COVID-19 epidemic in Henan Province were collected, namely, the number of new confirmed cases/cured and discharged cases, the cumulative number of confirmed cases/cured and discharged cases/deceased cases, and the number of severe and critical cases. Age, sex, incubation period, COVID-19 case source type, the number of new confirmed cases, the number of cured and discharged cases, and the number of deaths in each city were collected through the details of confirmed COVID-19 cases published on the official websites of the municipal health commissions.

### Data classification.

The data investigated in this paper include age, sex, incubation period, cumulative confirmed cases, cumulative cured and discharged cases, daily new cases/cured cases, number of permanent residents in Henan Province and the 6 cities, sex and age structures of Henan Province and the 6 cities, source of the confirmed cases, and types of confirmed cases. A suspected case was defined as an individual with fever, sore throat, and cough who had a history of travel to China, other areas of persistent local transmission, or contact with patients with similar travel histories or contact with confirmed COVID-19 cases. However, cases may be asymptomatic or even without fever (18). Confirmed cases were defined according to real-time PCR testing of respiratory or blood samples; the samples that tested positive for SARS-CoV-2 were considered confirmed cases (19). This article divides the case source type into three categories: imported cases, contact cases, and local cases (Table 4). The types of confirmed cases are divided into four categories: common (mild) cases, severe cases, critical cases, and fatal cases (Table 5). The calculations for the statistics presented are as follows: cumulative incidence of COVID-19 (1/100,000) = cumulative confirmed cases of COVID-19 in a city (county included)/permanent resident population in a city at the end of 2018; cumulative cure rate (%) = cumulative number of cured and discharged COVID-19 cases in a city/cumulative number of confirmed COVID-19 cases in a city; cumulative mortality rate (%) = number of COVID-19 deaths in a city/number of COVID-19 confirmed cases in a city.

**TABLE 4:** Source of confirmed COVID-19 cases

Type	Definition
Imported cases	The patient had a history of overseas travel before diagnosis
Contact cases	Contact with confirmed or suspected patients before diagnosis
Local case	The patient had not left the city before diagnosis and had no contact history

**TABLE 5:** Types of confirmed cases of COVID-19^*a*^

Type	Characteristic feature(s)
Common (mild) cases	Mild clinical symptoms, visible imaging/no manifestations of pneumonia
Severe cases	In addition to the characteristics of the common cases, oxygen saturation ≤93% under resting state, partial arterial oxygen pressure (PaO_2_)/oxygen absorption concn (FiO_2_) ≤300 mm Hg, and pulmonary imaging showing significant lesion progression of more than 50% within 24 to 48 h
Critical cases	Patients have symptoms of respiratory failure or shock, require mechanical ventilation, or have multiple organ failure requiring ICU monitoring and treatment
Deceased cases	Death

a ICU, intensive care unit.

### Analytical methods.

Due to limited information on confirmed COVID-19 cases published on the official websites of the health commissions in the selected cities, a total of 600 cases in 4 cities (Zhengzhou, Xinyang, Nanyang, and Puyang City) with comprehensive information were selected for statistical analysis. Microsoft Office Excel 2016 was used for data collation and analysis. The maximum value, minimum value, and mean value were used to describe the measurement data, and the selection rate and proportion were used to describe the count data. Statistical charts and tables were used to describe the overall trend, sex and age distribution ratio, case source type, and case type composition ratio of the COVID-19 epidemic in Henan Province and the 6 selected cities.
